# Electrophysiological monitoring of the nonrecurrent inferior laryngeal nerve and radiological evaluation of concurrent vascular anomalies

**DOI:** 10.3389/fendo.2024.1420697

**Published:** 2024-09-20

**Authors:** Ismail Cem Sormaz, Ahmet Yalin Iscan, Fatih Tunca, Mehmet Kostek, Nurcihan Aygun, Tugba Matlim Ozel, Yigit Soytas, Arzu Poyanli, Serkan Sari, Mehmet Uludag, Yasemin Giles Senyurek

**Affiliations:** ^1^ Division of Endocrine Surgery, Department of General Surgery, Istanbul Faculty of Medicine, Istanbul University, Istanbul, Türkiye; ^2^ Division of Endocrine Surgery, Department of General Surgery, Sisli Hamidiye Etfal Training and Research Hospital, University of Health Sciences Turkey, Istanbul, Türkiye; ^3^ Division of Endocrine Surgery, Department of General Surgery, Basaksehir Cam and Sakura City Hospital, University of Health Sciences Turkey, Istanbul, Türkiye; ^4^ Department of Radiology, Istanbul Faculty of Medicine, Istanbul University, Istanbul, Türkiye

**Keywords:** nonrecurrent inferior laryngeal nerve, arteria lusoria, intraoperative nerve monitoring, aberrant right subclavian artery, Kommerell diverticulum, abnormal vertebral artery

## Abstract

**Purpose:**

The objective of this study was to characterize the electrophysiological characteristics of nonrecurrent inferior laryngeal nerves (NRILNs) that were dissected via intraoperative neuromonitoring (IONM) and concomitant vascular anomalies in patients with NRILNs.

**Methods:**

A retrospective analysis was conducted on 7865 patients who underwent thyroidectomy with IONM at three tertiary referral centers. The study included 42 patients in whom an NRILN was detected. IONM data and postoperative vocal cord (VC) examinations were recorded for all patients. The absence of an initial vagal EMG response and/or a short (<3.5 ms) latency period during the initial vagal stimulation or the inability to identify the RLN within the Beahrs triangle was considered highly suspicious for the presence of an NRILN. Postoperative cross-sectional imaging was performed in 36 out of 42 patients to assess any concurrent vascular anomalies.

**Results:**

The prevalence of NRILN was 0.53%. An NRILN was suspected due to EMG findings in 32 (76%) patients and the inability to identify the RLN within the Beahrs triangle in the remaining 10 (24%) patients. The mean right VN latency period was 3.05 ± 0.15 ms. The V1 latency period of the right VN was shorter than 3.5 ms in 39 (93%) and longer than 3.5 ms in 3 (7%) patients. One of these three patients with latency>3.5ms had a large mediastinal goiter. Transient VC paralysis occurred in one (2.4%) patient. Of the 36 patients with postoperative imaging data, 33 (91.4%) had vascular anomalies. All 33 patients had aberrant right subclavian arteries, and 13 (39.4%) also had accompanying additional vascular anomalies.

**Conclusion:**

The NRILN is an anatomical variation that increases the risk of nerve injury. Observation of an absent EMG response and/or a short latency period during the initial vagal stimulation facilitates the detection of an NRILN at an early stage of thyroidectomy in the majority of patients.

## 1 Introduction

The most common endocrine surgery procedure is thyroidectomy, which carries a potential risk for recurrent laryngeal nerve (RLN) injury (1). The presence of anatomical variations in the RLN might render the nerve more prone to injury. To decrease the rate of RLN injury during thyroidectomy, surgeons should be aware of anatomical variations, perform meticulous surgical procedures, and use essential auxiliary tools.

The nonrecurrent inferior laryngeal nerve (NRILN) is rare, and the incidence of this anatomical variation is greater on the right side (0.4 to 2.4%) than on the left side (0.004%) (1). On the left side, the NRILN is associated with accompanying anomalies, such as situs inversus, deletion of the ductus arteriosus, and the right aortic arch; these conditions are mostly fatal and render NRILN a very rare occurrence (2). In the right-sided NRILN, concurrent vascular anomalies, such as the absence of a brachiocephalic artery, a common carotid artery originating directly from the aortic arch, and an aberrant right subclavian artery, were also observed (3).

Branching of the RLN is a common anatomic variation of the RLN (4, 5), but the presence of the NRILN can lead to a serious risk of nerve injury during thyroidectomy. In patients with NRILN vocal cord paresis, up to 75% of cases may occur (6). The course of the NRILN from the vagus nerve to the larynx is variable. Although routine visual identification of the RLN is crucial for a thyroid surgeon, the use of intraoperative neuromonitoring (IONM) facilitates mapping of the course of the nerve, helps surgeons recognize anatomical variations, and provides real-time functional information. Proper use of IONM helps to identify anatomical variations at an early stage of the operation and preserve the RLN (7).

In this article, we describe the electrophysiologic monitoring of the NRILN in 42 patients via standardized use of IONM during thyroidectomy and vascular anomalies concurrent with the NRILN detected by postoperative cross-sectional imaging (computerized tomography (CT) or magnetic resonance imaging (MRI) scans).

## 2 Patients and methods

This retrospective study was conducted at three tertiary referral centers of endocrine surgery, namely, the Istanbul Faculty of Medicine of Istanbul University (Center 1), the Istanbul Şişli Hamidiye Etfal Training and Research Hospital of University of Health Sciences (Center 2) and the Turkish Ministry of Health Basaksehir Cam and Sakura City Hospital of the University of Health Sciences(Center 3).

The data of 8172 patients who underwent thyroid surgery between January 2016 and June 2023 were retrospectively reviewed. Of these 8172 patients, 307 were excluded due to incomplete data. The remaining 7865 patients were retrospectively analyzed to evaluate the incidence of NRILN. Of the 7865 operations, 3018 (38.4%), 2954 (37.5%) and 1893 (24.1%) were performed in Centers 1,2 and 3, respectively (**Table 1**). All thyroid operations were performed by using either continuous (C-IONM) or intermittent intraoperative neuromonitoring (I-IONM).

**Table 1:** Patient distribution and NRILN rates among three centers.

	Number of Patients (n,%)	NRILN (n,%)
Center 1	3018, 38.4%	18, 0.6%
Center 2	2954, 37.5%	12, 0.4%
Center 3	1893, 24.1%	12, 0.6%
Total	7865	42

Of the 7865 patients included in this study, 42 (0.53%) had NRILNs. The rate of patients with NRILN was 0.6% (n=18), 0.4% (n=12), and 0.6% (n=12) in Centers 1, 2, and 3, respectively. The data of these 42 patients, including age, sex, preoperative thyroid function test results, thyroid ultrasonography and/or thyroid scintigraphy results, pre- and postoperative vocal cord examination results with indirect laryngoscopy, surgical indications, surgical extent, intraoperative IONM data and postoperative histopathological diagnosis, were analyzed retrospectively.

These 42 patients were recalled after surgery for contrast or noncontrast CT or MRI scans of cervical and thoracic regions to reveal any vascular anomalies concurrent with the NRILN. A noncontrast CT scan was performed for patients with known contrast media allergy or chronic renal insufficiency. IONM records were available for all 42 patients with NRILN. Postoperative cross-sectional imaging was obtained for 36 patients but could not be performed for the remaining 6 patients. Of these six patients, 4 refused to undergo postoperative imaging, 1 died due to a disease unrelated to thyroid surgery, and 1 had moved abroad. Cross-sectional scan images were analyzed by an experienced radiologist (AP).

This study was approved by the Ethics Committee of Istanbul University, Istanbul Faculty of Medicine (No. 2358861). Informed consent was obtained from each patient before undergoing cross-sectional imaging. The patients were informed that the imaging would be performed for descriptive purposes and would not affect the future management of their disease.

### 2.1 IONM Set-up and anesthesia protocol

Equipment setup, anesthesia, applications, IONM standards, EMG modality parameters, and interpretation of IONM data were performed according to the guidelines of the International Intraoperative Neural Monitoring Study Group (INMSG) (8). All NARs were dissected by using continuous or intermittent IONM, including EMG data at the beginning and at the end of the operation and preoperative and postoperative laryngoscopic examination (L1, V1, R1, R2, V2, L2) according to the recommendations of the INMSG (8). IONM was performed by using one of the following two commercially available neuromonitoring devices: (1) an NIM 3.0 Nerve Monitoring Systems (Medtronic Xomed, Jacksonville, FL, USA) or (2) an Avalanche SI (Dr. Langer Medical GmbH, Waldkirch, Germany).

### 2.2 Thyroid surgery and intraoperative nerve monitoring technique

Initial vagal neurostimulation was performed at the level of the cricoid cartilage or inferior thyroid artery (ITA) according to the preference of the surgeon. A V1 amplitude of more than 500 microvolts (µV) was considered ideal, and an amplitude of more than 300 µV was considered acceptable for proceeding with the operation. After achieving the appropriate V1 amplitude, the vagal stimulation probe was gently placed on the vagus nerve (VN) in patients for whom C-IONM was performed.

The RLN was first identified with a neuromonitoring hand probe and then visually confirmed within Beahr’s triangle, which is bordered by the common carotid artery as the base, the ITA superiorly, and the RLN as the third side (9). The RLN dissection was continued cranially along its course to the laryngeal entry point. All operations were performed by experienced endocrine surgeons at all three centers participating in the study.

The absence of a V1 EMG response or a short (<3.5 ms) latency period of the EMG wave obtained during the initial stimulation of the VN or the inability to identify the RLN by the neuromonitoring hand probe and visually within the Beahrs triangle was considered to indicate the presence of an NRILN (10). When NRILN was suspected, the VN was stimulated from the level of the superior border of the thyroid cartilage down to the level of the clavicle. The observation of a positive EMG response at the proximal level but no EMG response at the distal level of the VN was considered indicative of the NRILN (11). The degree to which the EMG response on the VN was lost was determined, and an NRILN was sought at this level by using a neuromonitoring hand probe before visual identification of the nerve. After visual identification of the NRILN, the nerve was dissected from the point where it separated from the VN along its course to the laryngeal entry.

When C-IONM was used in patients with NRILN, the vagal stimulation probe was placed on the proximal segment of the VN where a positive EMG response was present. Depending on the origin point of the NRILN insertion of the vagus electrode to the VN, it may be technically difficult. In such cases, where the junction of the VN and the NRILN is located upwards, the thyroidectomy incision was extended laterally. The carotid sheath was meticulously dissected up to the branching point of the NRILN from the VN, and the vagus electrode was placed proximal to this point. In patients who underwent lateral neck dissection, an extension of the incision was not required.

The right V1 latency was calculated as the duration between the stimulation spike and the initial deflection of the evoked waveform from the zero baseline.

### 2.3 Classification of the NRILN

The course of the NRILN from the VN to the laryngeal entry was evaluated, and the type of NRILN was defined according to the classification described by Toniato et al. (12). The NRILN was defined as Type 1 when the nerve separated directly from the cervical VN and followed a course along with the vessels of the upper thyroid stalk; Type 2A when the NRILN showed a transverse path parallel to and over the trunk of the ITA; and Type 2B when the NRILN was found below the trunk or between the branches of the ITA.

## 3 Results

### 3.1 Preoperative results

The mean age of the 42 patients with NRILNs was 46.8 ± 11 years, with a predominance of female patients (%93). The indications for thyroidectomy included indeterminate, suspicious for malignancy or malignant cytology in 17 (40.5%) patients; large goiter with compressive symptoms in 15 (35.7%) Basedow Graves disease in 5 (11.9%) patients; and multinodular toxic goiter in 5 (11.9%) of 42 patients.

None of the 42 patients underwent cross-sectional imaging preoperatively.

### 3.2 Intraoperative findings

Of the 42 patients, total thyroidectomy was performed in 30 (71.4%), total thyroidectomy and central lymph node dissection were performed in 7 (16.7%), and right lobectomy was performed in 5 (11.9%) patients.

C-IONM or I-IONM was used in 20 (47.6%) or 22 (52.3%) patients, respectively. Of the 42 patients, 32 (76%) were suspected to have an NRILN due to intraoperative EMG findings (absence of initial EMG response on the VN or short latency period), and 10 (24%) patients were unable to identify the RLN within the Beahrs triangle. All 42 NRILNs were found on the right side.

The presurgical dissection and postsurgical dissection amplitude and latency values for the right VN and the right NRILN are summarized in **Table 2**.

**Table 2:** Presurgical dissection and postsurgical dissection amplitude and latency mean values of the right VN and the NRILN.

	V1	V2	R1	R2
Amplitude (µV) Mean ± SD Range	741 ± 446 380-1800	1217 ± 701 252-2700	992 ± 592 170-2200	1097 ± 772 250-3900
Latency (ms) Mean ± SD Range	3.05 ± 0.15 1.5-5.1	3.06 ± 0.14 1.5-3.25	2.34 ± 0.11 1.4-3.25	2.44 ± 0.12 1.4-3.25

V1: Presurgical dissection amplitude/latency values of the vagus nerve; V2: Postsurgical dissection amplitude/latency values of the vagus nerve; VN, R1: Presurgical dissection amplitude/latency values of the nonrecurrent inferior laryngeal nerve; R2: Postsurgical dissection amplitude/latency values of the nonrecurrent inferior laryngeal nerve.

Of the patients with NRILN, the initial latency period of the right VN was shorter than 3.5 ms in 39 (93%) and longer than 3.5 ms in the remaining 3 (7%) patients. One of the three patients with a latency period longer than 3.5ms had a large goiter extending retrosternally on the right side, with a V1 latency of 5.1ms. Furthermore, this patient was found to have an accompanying aberrant subclavian artery on cross-sectional imaging. Two patients, both with a latency exceeding 3.5 ms, underwent surgery for papillary thyroid cancer. In one case, a 1.5 cm papillary thyroid cancer was identified in the right lobe along with lateral cervical lymph node metastasis. The dimensions of the right lobe were measured at 5x3.5x2 cm. The V1 latency period in this patient was recorded at 4.4 ms, and postoperative imaging revealed the presence of a coexistent aberrant subclavian artery. In the second case with PTC and long latency period, the dimensions of the right lobe were recorded as 4x2x2 cm. The V1 latency in this patient was 3.8 ms; however, assessment for any vascular anomalies was hindered by the lack of postoperative cross-sectional imaging availability.

There were 14677 nerves at risk (NAR) in 7865 patients. Of the 14677 NARs, 7385 (50.4%) were located on the right side and 7292 (49.6%) on the left side. All 42 detected NRILNs were identified on the right side. The incidence of NRILN was 0.53% in 7865 patients, 0.28% in all 14677 NARs, and 0.56% in 7385 right-sided NARs. No NRILN was found on the left side.

**Figure 1:**
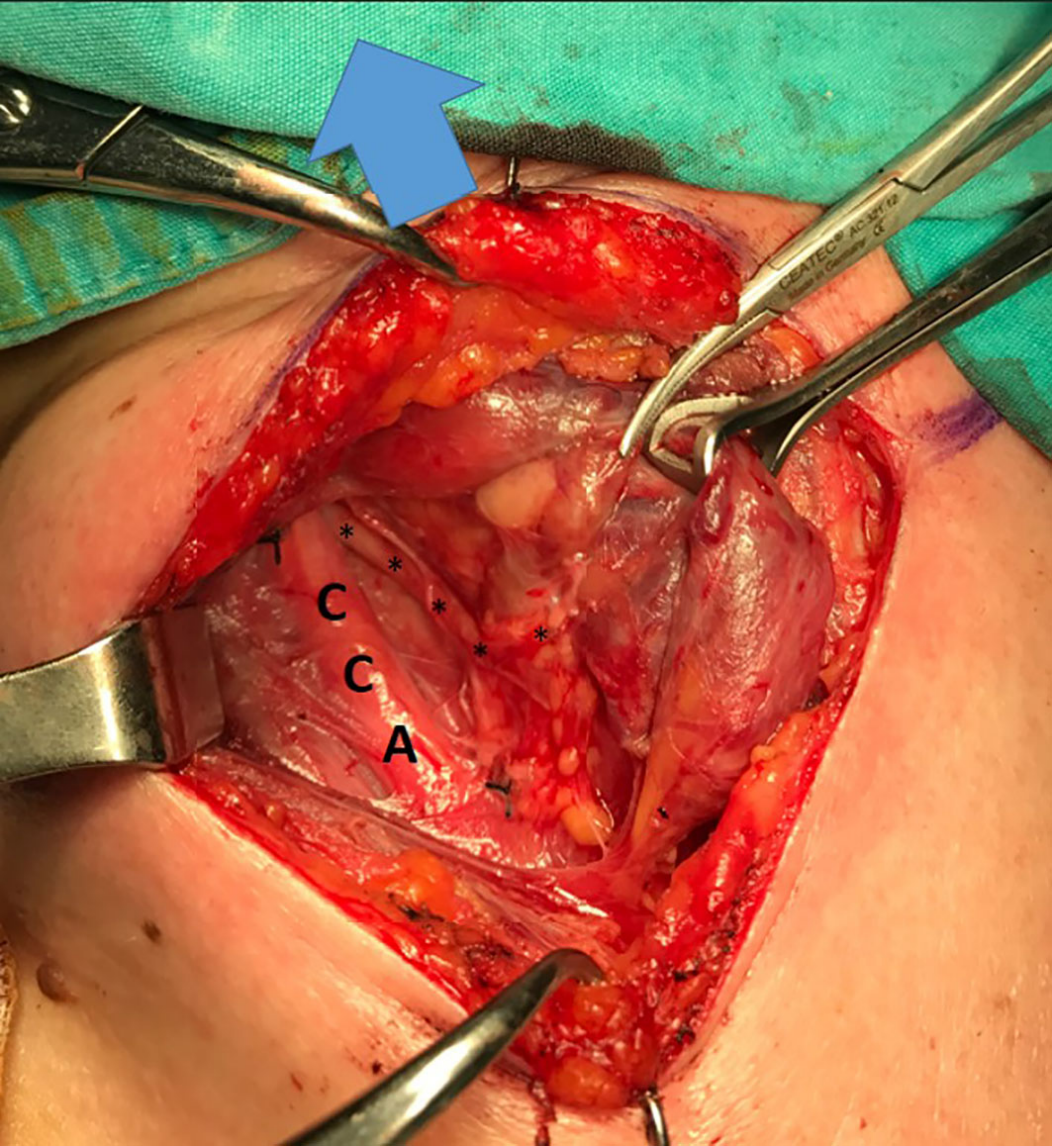
Type 1 NRILN, arrow: cranial side; CCA, common carotid artery; trace of type 1 NRILN shown with (↓).

**Figure 2:**
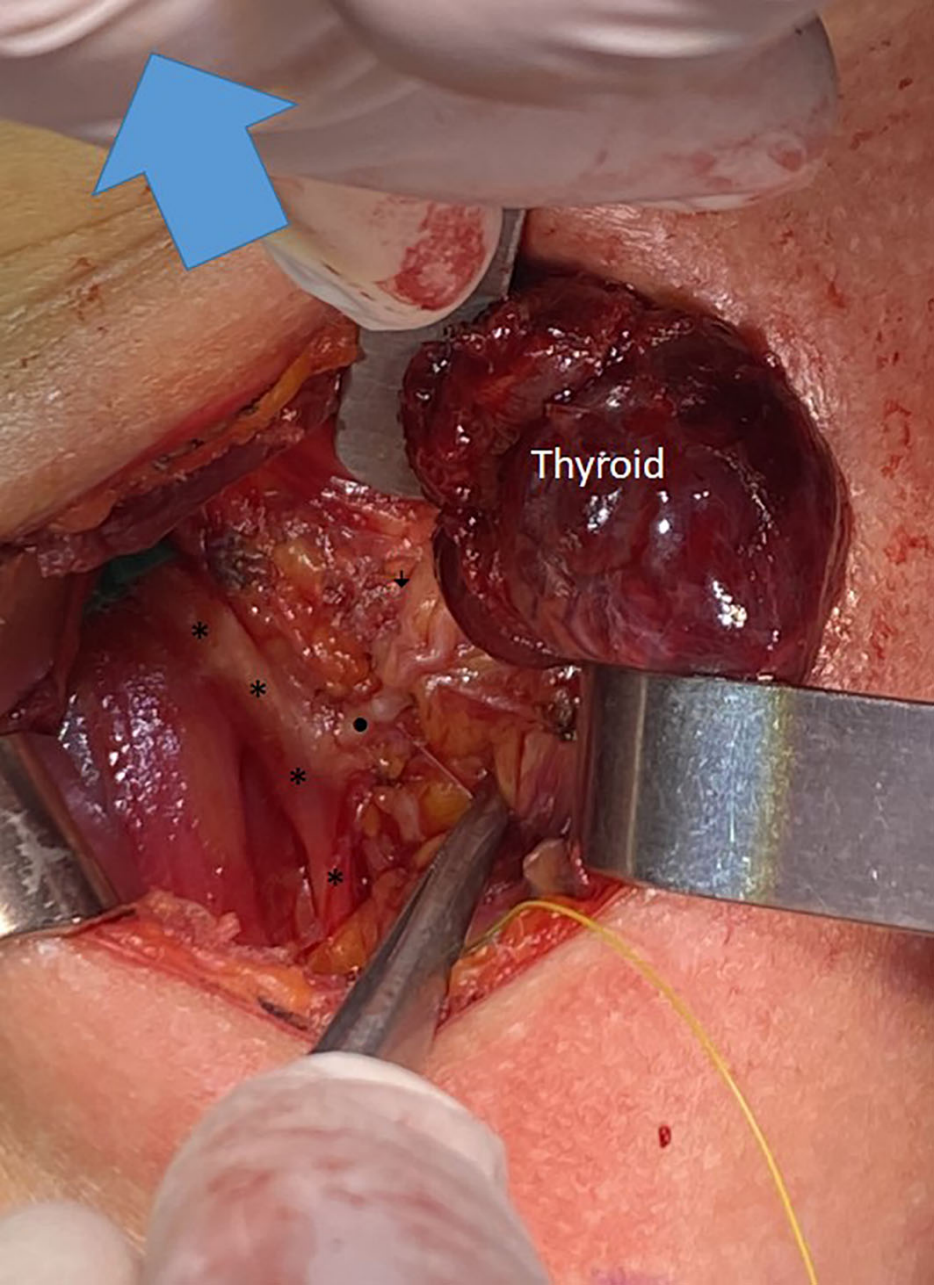
A type 2A NRILN showing extralaryngeal branching; the main trunk of a type 2A NRILN (•) originates from the vagus nerve (*). The NRILN showed extralarengeal branching. The (↓) points to the posterior branch with no EMG response.

**Figure 3:**
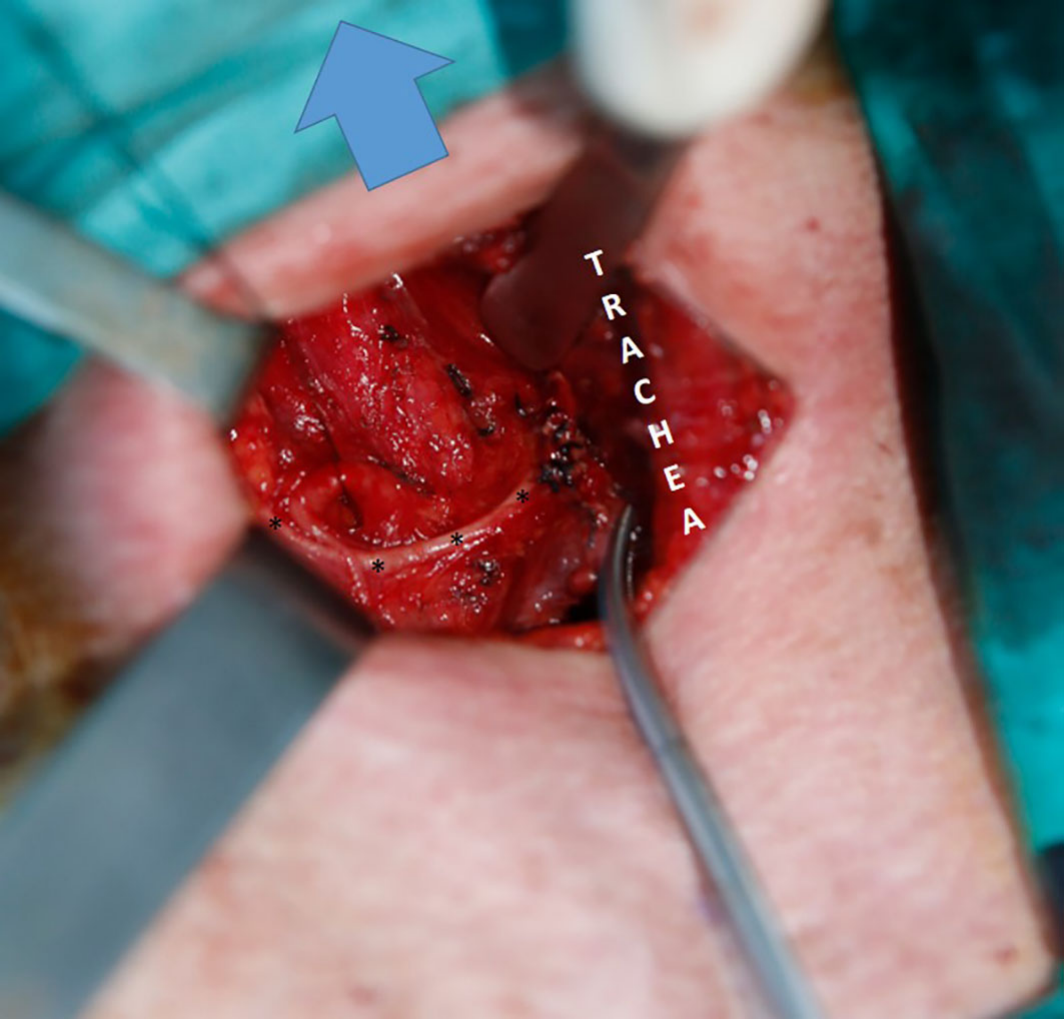
Type 2B NRILN; arrow: Cranil side, ITA suspended with a silk suture; trace of type 2B NRILN shown with (*).

Of the 42 NRILNs, 10 (24%), 29 (69%), and 3 (7%) were classified as type 1, type 2A and type 2B, respectively (**Figures 1**–**3**).

Intraoperative LOS occurred in 1 (2.4%) of the 42 patients. C-IONM was used in this patient, and a type 1 NRILN was identified by IONM before nerve dissection on the second side of thyroidectomy. However, LOS can occur due to unavoidable traction injury.

### 3.3 Postoperative results

Histopathologic examination revealed follicular nodular disease in 22 (52.4%) patients and papillary thyroid carcinoma in 20 (47.6%) patients (including 5 papillary microcarcinoma patients). One (2.4%) patient with type 1 NRILN, in whom LOS occurred during thyroidectomy, had transient vocal cord paralysis that resolved within 3 months after surgery.

### 3.4 Postoperative imaging

**Figure 4:**
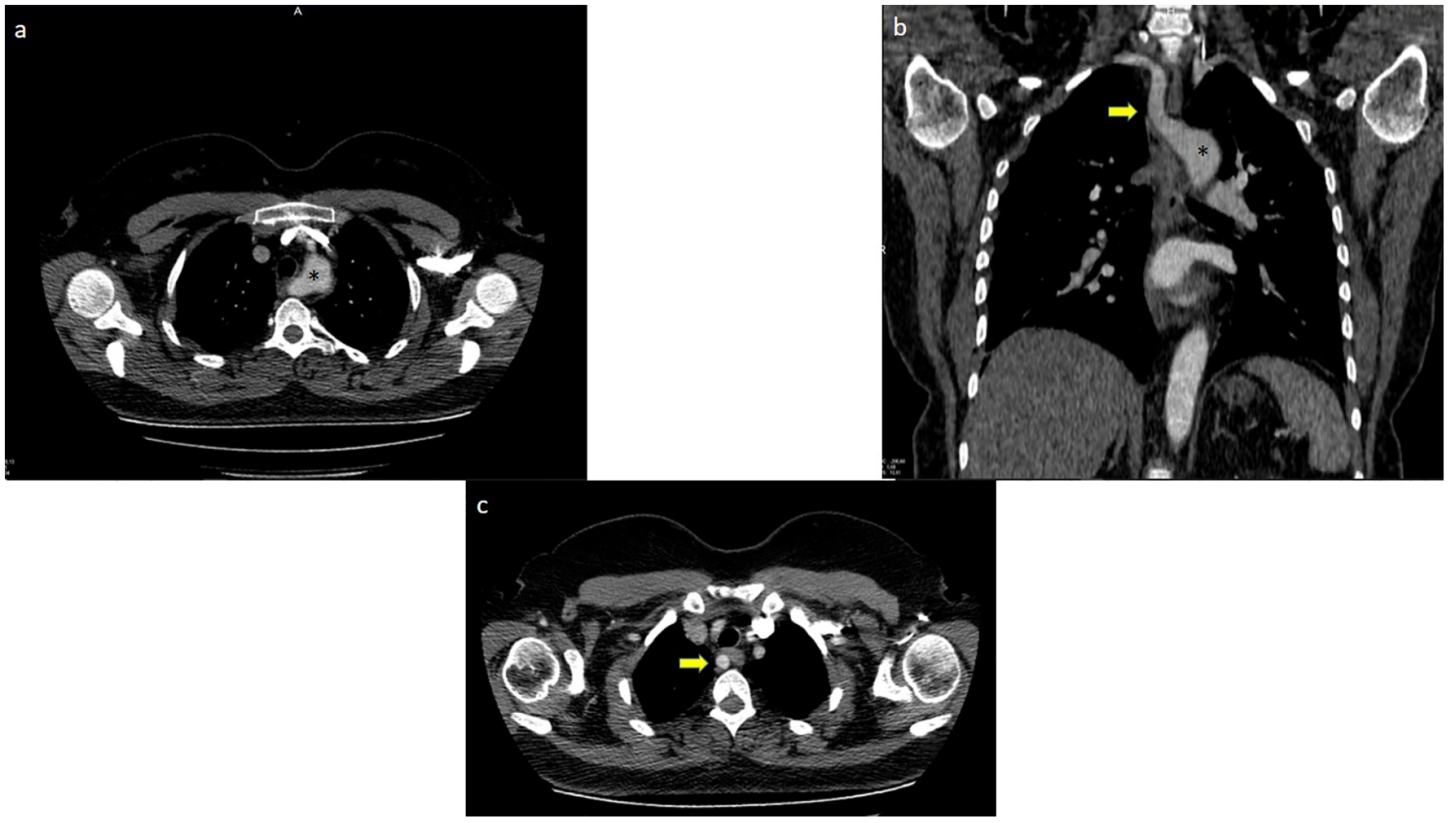
Aberrant right subclavian artery +Kommerel Diverticulum; **(A)** (*) points Kommerel Diverticulum, **(B)** (*) points Kommerel Diverticulum, arrow points Aberrant Right Subclavian Artery, **(C)** Arrow points Aberrant Right Subclavian Artery.

**Figure 5:**
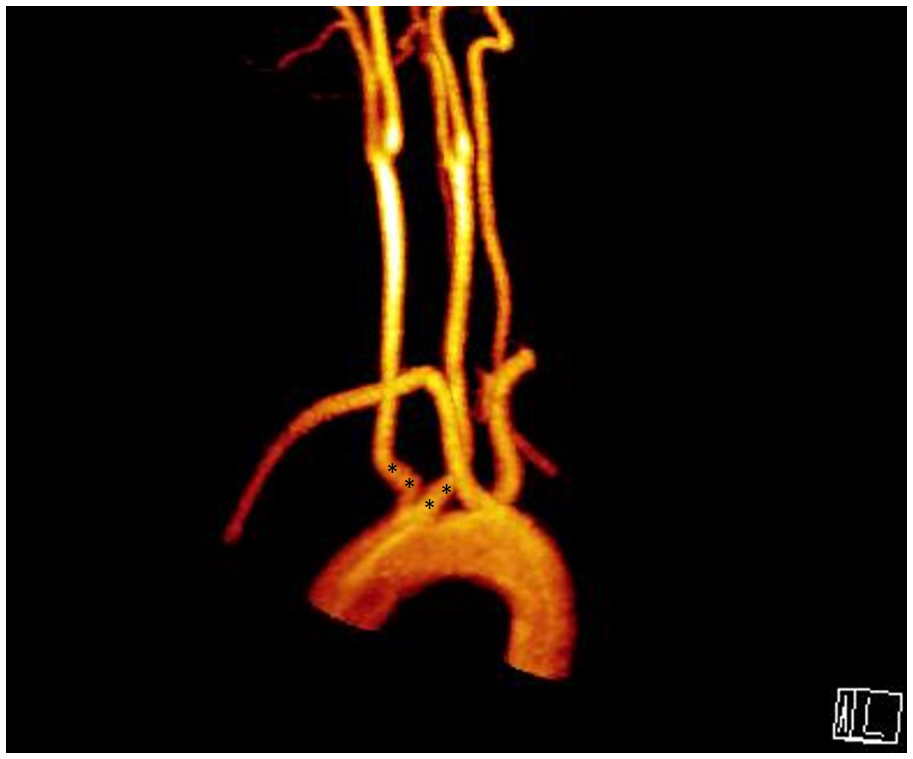
Aberrant right subclavian artery + bicarotid truncus; (*) points root of bicarotid truncus.

**Figure 6:**
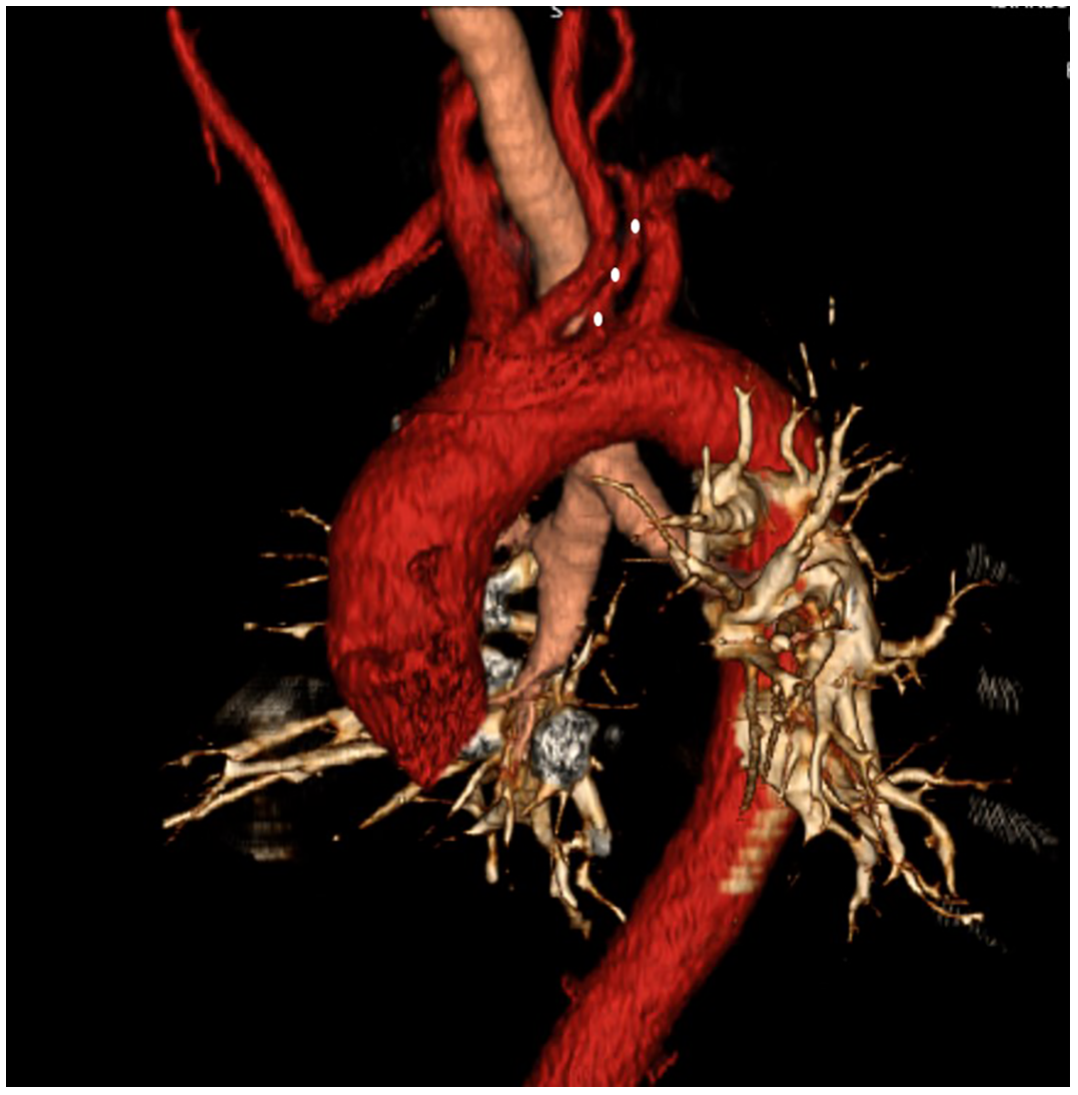
Aberrant right subclavian artery + left vertebral artery originating from the arcus aorta (white dots).

Four different types of vascular anomalies, including aberrant right subclavian artery (Arteria Lusoria), Kommerell diverticulum, bicarotid truncus, and abnormal vertebral artery, were identified by cross-sectional imaging in 33 (91.4%) of 36 patients. All 33 patients had aberrant right subclavian arteries. Of these 33 patients, 13 (39.4%) had accompanying additional vascular anomalies, including Kommerell diverticulum in 6 (46%), bicarotid truncus in 4 (30.8%), Kommerell diverticulum + bicarotid truncus in 2 (15.4%), and AVA in 1 (7.8%) (**Figures 4**–**6**).

## 4 Discussion

One of the most feared complications of thyroidectomy is RLN injury, with temporary unilateral RLN injury occurring in 1% to 20% of cases but usually occurring at a rate of 1% to 2% in high-volume centers (13). According to a meta-analysis of 25000 patients, the rates of transient and permanent RLN palsy vary from 1.4% to 38.4% (with an average of 9.8%) and from 0% to 18.6% (with an average of 2.3%), respectively (14). In a comparative study by Dralle et al., primary cancer surgery, revision surgery for benign goiter or malignancy, lobectomy *vs* subtotal resection, low hospital and surgeon volume, and no RLN identification during thyroidectomy were found to be risk factors for postoperative permanent RLN palsy (15). Identifying the RLN and dissecting the nerve till its laringeal entry point are the most effective methods for preventing nerve injury and reducing the incidence of RLN palsy (9). When the RLN is properly identified, the risk of injury is minimized to 0-2.1%, but the risk can increase to 4-6% if the nerve is not dissected properly (16). The introduction of IONM during thyroidectomy enabled us to assess the functional integrity of the RLN. Furthermore, IONM provides extensive knowledge about the mechanisms of RLN injury and the anatomical variations that cause the RLN to be prone to injury. A recent meta-analysis including randomized clinical trials demonstrated an absolute reduction in overall RLN injury of 1% and a relative reduction of more than 25% by using IONM compared to only visual identification of the RLN, although the results failed to achieve statistical significance (17).

The anatomy of the RLN can be challenging due to anatomical variations, such as extralaryngeal branching, distortion of the nerve due to a large substernal goiter or large recurrent goiter, intertwining branches of the RLN and ITA, and the presence of the nonrecurrent inferior laryngeal nerve (18). The application of IONM during thyroidectomy helps early identification of these anatomical variations (19).

With an incidence of 0% to 4.76%, NRILN is a rare occurrence that is typically located on the right side. In rare cases, NRILN may appear on the left side (0.04%) (20). Preoperative determination of this variation is rare; therefore, intraoperative recognition of the NRILN is very important for avoiding nerve injury. The presence of the NRILN is associated with an increased risk of nerve injury and vocal cord palsy (4, 12). Iacobone et al. reported a significantly greater risk of vocal cord palsy in patients with an NRILN than in those with a normal RLN (14.3% *vs*. 1.75%) (4). Toniato et al. retrospectively analyzed the data of 6000 thyroidectomies performed during a 20-year period and observed right-sided NRILNs in 31 (0.51%) patients (12). The authors performed routine RLN identification and reported injury rate of 12.9% in patients with NRILN, which was much greater than the 1.8% in those who had RLN with a normal course. In these two studies by Iacobone et al. and Toniato et al., IONM was not used during thyroidectomy.

In 2002, Brauckhoff et al. described a method of selective neurostimulation of the vagus nerve to recognize the NRILN early during thyroidectomy (21). The authors concluded that observation of a negative response by neurostimulation of the VN distally at the level of the lower thyroid pole and a positive EMG response proximally at the level of the upper thyroid pole necessitated the search for an NRILN. The authors identified all nine patients with NRILN by using the described neurostimulation method.

Wang et al. investigated the accuracy of methods for predicting the presence of an NRILN by using IONM in 73 NRILNs (11). The authors defined two specific points on the VN: point A, at the superior border of the thyroid cartilage, and point B, at the inferior border of the fourth tracheal ring. A positive EMG signal at point A and a negative signal at point B indicated the presence of an NRILN. Comparison of points A-B revealed that the EMG response accurately predicted the NRILNs in 97.2% of the patients. They concluded that a comparison of points A-B combined with the observation of low latency period achieved 100% sensitivity and specificity in predicting the NRILN. The contribution of IONM to the exploration and preservation of the RLN is undeniable, especially in cases with anatomical variations, especially when the NRILN is considered a rare variation (13–15). Donatini et al. observed an increased detection rate of NRILN by routine use of IONM during thyroidectomy and reported a decreased rate of nerve injury, especially in patients with NRILN (22). The authors mentioned that no nerve injury occurred in any of the 11 NRILNs dissected by using IONM.

There are several types of NRILNs classified according to their nerve travel pattern (2, 12, 20). In our study, we used the preferred Toniato classification system to classify NRILNs (12). We found that 69% of the NRILNs in our patients were type 2A (69%), and these findings are consistent with the literature (12, 20).

Among type 1 NRILNs, the nerve might be more prone to injury during upper pole dissection due to its greater proximity (23). However, in the study by Brauckhoff et al., all of the NRILNs were type 1, and no nerve injury occurred during thyroidectomy, as NRILNs were detected early during surgery via neurostimulation (21). In our study, intraoperative LOS and transient vocal cord palsy occurred in one patient with type 1 NRILN, although the nerve was identified by IONM before nerve dissection.

In our patients, the majority (76%) of the NRILNs were suspected to be present before nerve dissection due to either the absence of a V1 EMG signal or a V1 latency value lower than 3.5 ms.

In a previous study from our institution, the mean value of V1 latency of the right VN was found as 4.8 ± 1.0 in our patient population (24). In the present study, the mean V1 latency of the right VN was 3.4 ms. As formerly emphasized by Brauckoff et al., a low V1 latency value (<3.5 ms) was associated with the presence of an NRILN (10).

However, Brauckoff et al. presented a case with an NRILN in whom a V1 latency period longer than 3.5 ms was observed. This patient had a large recurrent mediastinal goiter. The right V1 latency period was found to be 3.3 ms when the VN was stimulated at the level of the ITA, and 3.75 ms when the nerve was stimulated at the level of the carotid artery bifurcation in this patient. The authors concluded that the V1 latency might be longer than 3.5 ms in such cases with a large goiter due to elongation of the NRILN (25). In a prospective multicenter study that investigated the normal quantitative parameters of IONM in 1996 NARs, showed that the right V1 latency ranged between 3.13 ms and 4.69 ms (26). Although a short V1 latency is highly suspicious for an NRILN, a longer latency period does not always exclude an NRILN. Several factors, such as the level of initial VN stimulation or elongation of the nerve due to mediastinal goiter might prolong the initial V1 latency period in patients with NRILN (25). Within our study, it was noted that a patient with a large goiter protruding into the retrosternal area on the right side exhibited a right V1 latency period of 5.1 ms.

The depiction of latency in monitoring studies has varied, lacking a universally accepted point on the waveform for measurement (8). In Brauckhoff et al.’s study, latency was characterized as the duration from the stimulation spike to the initial deflection of the evoked waveform from the zero baseline (10). Conversely, Kamani et al. measured latency as the interval from the stimulation spike to the first peak of the evoked waveform in their investigation outlining the electrophysiological parameters of the NRILNs (27). In our study, Vı latency was defined as the time from the stimulation spike to the initial deflection of the evoked waveform from the zero baseline.

In our patient cohort, three (7%) patients with NRILN exhibited V1 latency periods exceeding 3.5 ms, a rate relatively higher than reported in existing literature (10). In one of the three patients, the prolonged latency period may be attributed to nerve elongation caused by a large retrosternal goiter on the right side. However, in the other two patients (4.8% of the cohort), latency exceeding 3.5ms could not be accounted for by thyroid anatomical features or NRILN. The latency duration is influenced by the proximity of the stimulation site to the ipsilateral vocal cord (25). The long latency period in the latter two patients may be attributed to variations in the initial vagal stimulation method, which was conducted either at the level of cricoid cartilage or the ITA based on the surgeon’s preference.

In 24% of our patients, the presence of the NRILN was suspected because the RLN could not be detected visually or by a neuromonitoring hand probe in Beahr’s triangle, which emphasizes the importance of routine RLN identification during thyroid surgery.

In 1823, the NRILN on the right side with accompanying vascular anomaly (arteria lusoria) was first described by Stedman (28). As the presence of the NRILN results from anomalous embryonic development of the aortic arches, vascular anomalies are expected to accompany all patients with NRILN. However, a recent meta-analysis revealed that of the 136 right NRILN patients, 89.3% had concurrent aberrant subclavian arteries (29). In a study by Rafaelli et al., 656 right inferior laryngeal nerves (ILNs) were dissected, and an NRILN was found in three (0.46%) patients, all of whom had accompanying aberrant subclavian arteries (30). However, the authors reported large anastomotic branches between the cervical sympathetic chain and inferior laryngeal nerve (SILAB), mimicking the NRILN on the right side in 10 (1.5%) patients, and none of these patients had concurrent vascular anomalies. They concluded that when these anastomotic branches are as large as the ILN, they could be mistaken for a ‘false’ nonrecurrent ILN visually. Another study by Parpounas et al. described a method to recognize the SILAB and avoid misinterpretation of these branches as NRILNs by using IONM in 133 patients (31). The authors initially stimulated the ipsilateral VN from the level of the larynx down to the clavicle and excluded the possibility of NRILN if there was a positive EMG response. In patients with a positive vagal EMG response at all levels, the presence of a structure mimicking the NRILN was described as SILAB when an RLN with a normal course was identified both visually and by using IONM. A right NRILN was found in one (0.75%) patient. The prevalence of right SILABs was 14.14%, and 21.4% of the SILABs had a diameter similar to that of the right RLN. The authors mentioned that no EMG response was detected by stimulation of the SILAB as SILAB does not provide motor stimulus to the larynx whereas positive EMG response was obtained in the patient with NRILN. In our study, IONM was used in all patients, and a positive EMG response was achieved by stimulating the NRILN in all patients.

In the present study of 36 patients with postoperative cross-sectional imaging, 33 (91.4%) patients had vascular anomalies concurrent with the NRILN. All 33 patients exhibited an aberrant right subclavian artery, 13 of whom also presented with other vascular anomalies.

Aberrant or variant vascular anatomy is not uncommon in clinical practice, with the incidence of aberrant subclavian arteries ranging from 0.5% to 2%. More than 20% of patients also present with other coexistent anomalies, such as chromosomal anomalies, right aortic arch (RAA) with mirror image branching (RAA-mirror), and extracardiac anomalies (32–34). Kommerell’s diverticulum, a diverticulum at the origin of the aberrant right subclavian artery, has a broad base and is formed by a persisting right aortic arch (35, 36). Epstein and DeBord reported that 60% of aberrant subclavian arteries are present alongside Kommerell’s diverticulum (32). Preoperative cross-sectional imaging of the neck may help to predict NRILN through identification of the aberrant right subclavian artery (37, 38). In the study by Gao et al., which included 1574 consecutive thyroid cancer patients who underwent preoperative CT scans, the presence of an NRILN was preoperatively predicted in 7 of 9 patients with NRILN (37). The NRILN was recognized intraoperatively by IONM in the remaining two patients who were found to have aberrant right subclavian arteries on retrospective analysis of CT images.

It is not feasible to perform routine cross-sectional imaging only to predict the presence of an NRILN, which has a very low prevalence. Routine use of IONM during thyroidectomy enables safe recognition of an NRILN (39).

The other method described for the preoperative prediction of NRILN involvement is visualization of the aberrant right subclavian artery via neck ultrasonography (US). Iacobone et al. conducted a thorough preoperative US in 878 patients to identify the bifurcation of the brachiocephalic artery into the right common carotid and the right subclavian artery, also known as the Y sign (3). Patients with the Y sign detected by the US were considered to have normal vascular anatomy. Of the 37 patients without a Y-sign on the preoperative US, 17 (45.9%) had NRILNs identified intraoperatively. The authors concluded that the absence of the Y-sign by the US could predict the NRILN with an overall accuracy of 97.8%, and the detection of the common carotid artery directly originating from the aortic arch could predict the NRILN with an overall accuracy of 98.9%. Several other studies have also demonstrated that the sensitivity of the US to predict an NRILN ranged between 85-100% (6, 40). The US is a radiation-free, noninvasive, and cost-effective method that is part of preoperative evaluation before thyroidectomy. However, the US is an operator-dependent imaging modality, and patient-related factors such as obesity are important limitations of the US. Therefore, experienced radiologists are needed to identify vascular anomalous features on the neck US (41).

This study has several limitations. This was a retrospective multicenter study, and even though all thyroid surgeries were performed by experienced endocrine surgeons using systematic IONM, the initial vagal neurostimulation was not standardized. Surgeons performed initial vagal neurostimulation at either the cricoid cartilage or inferior thyroid artery based on their preference. These points could explain why only 76% of NRILNs were identified by using the IONM criteria and initial vagal latency was longer that 3,5ms in 7% of the patients with NRILNs. Postoperative cross-sectional imaging was able to be performed in 85.7% of patients with NRILN; however, it would be ideal for all patients to receive cross-sectional imaging.

## 5 Conclusion

The NRILN is a rare variable, and its preoperative detection is uncommon. The systematic and standardized use of IONM aids in identifying the NRILN early during thyroidectomy, helping patients avoid nerve injury. The absence of an EMG response and/or a short latency period (<3.5 ms) during initial vagal stimulation can help detect NRILN at an early stage of thyroidectomy in most patients. However, it should be kept in mind that a V1 latency longer than 3.5 ms does not always exclude an NRILN, and a considerable number of patients with an NRILN may have normal intraoperative EMG values which emphasizes the importance of routine anatomical identification of the RLN during thyroid surgery.

However, it should be kept in mind that a V1 latency longer than 3.5 ms does not always exclude an NRILN. Factors such as the presence of a large retrosternal goiter elongating the nerve and the level of initial vagal stimulation may lead to V1 latency periods exceeding 3.5 ms in patients with NRILNs. In such instances, patients with NRILN may present with normal intraoperative EMG values, highlighting the critical importance of consistently identifying the anatomical pathway of the recurrent laryngeal nerve during thyroid surgery.

## Data Availability

The raw data supporting the conclusions of this article will be made available by the authors, without undue reservation.
